# Clinical Outcomes and Optical Bench Analysis of a Novel Enhanced Monofocal Intraocular Lens

**DOI:** 10.3390/life15060984

**Published:** 2025-06-19

**Authors:** Giovanni Romualdi, Matilde Buzzi, Pier Giuseppe Ruggeri, Federico Tommasi, Alessio Giorgetti, Stefano Cavalieri, Rita Mencucci

**Affiliations:** 1 Eye Clinic, Department of Neuroscience, Psychology, Pharmacology and Child Health (NEUROFARBA), University of Florence, Largo Brambilla 3, 50134 Florence, Italy; matilde.buzzi@gmail.com (M.B.); piergiuseppe.ruggeri@unifi.it (P.G.R.); rita.mencucci@unifi.it (R.M.); 2 Department of Physics and Astronomy, University of Florence, via Giovanni Sansone 1, 50019 Sesto Fiorentino, Italy; federico.tommasi@unifi.it (F.T.); alessio.giorgetti@unifi.it (A.G.); stefano.cavalieri@unifi.it (S.C.)

**Keywords:** cataract surgery, IOL, enhanced IOL, EDOF IOL, Evolux, halos, optical quality

## Abstract

**Purpose:** A novel enhanced monofocal intraocular lens (IOL) has been developed to improve functional intermediate vision, maintaining a distance vision comparable to a standard monofocal lens and avoiding the drawbacks of multifocal IOLs. The aim of this study is to perform optical bench analysis and to evaluate refractive and visual outcomes and patient satisfaction. **Methods**: This prospective comparative single-center study was conducted in Careggi Hospital, University of Florence (Italy). We included 100 eyes from 50 patients who underwent bilateral cataract surgery. One group received the standard monofocal Tecnis GCB00 IOL, while the other group received the novel enhanced monofocal Evolux IOL. We evaluated binocular visual and refractive outcomes at 6 months after surgery. Binocular defocus curves and contrast sensitivity (CS) were also assessed. Optical quality was also analyzed in terms of higher-order aberrations (HOAs), modulation transfer function (MTF), objective scatter index (OSI), Strehl ratio, effective lens position (ELP), and halo analysis. A Patient-Reported Spectacle Independence Questionnaire (PRSIQ) was performed to assess spectacle independence outcomes. Finally, we analyzed the optical bench of both lenses. **Results**: All eyes implanted with Evolux achieved excellent distance vision, comparable to that achieved with GCB00. Evolux showed better intermediate and near vision, without any loss of visual quality, contrast sensitivity, or the presence of halos and photic phenomena. The optical bench analysis confirmed the different optical properties of the two lenses and supported the behavior obtained with the clinical defocus curve. **Conclusions**: These preliminary results show good refractive accuracy and visual outcomes for the enhanced monofocal IOL Evolux after cataract surgery. Further studies are needed to confirm our findings in terms of the number of patients and the period of follow-up.

## 1. Introduction

Monofocal lenses are the most frequently implanted intraocular lenses (IOLs) in cataract surgery, because of their relatively low cost, excellent outcomes for distance visual performance, and low values of photic phenomena (glare, halos) compared to multifocal IOLs [1]. Nevertheless, monofocal IOLs have a very limited depth of field and thus do not provide the functional intermediate and near vision required for most daily activities, which nowadays involve tablets, smartphones, and computers [2,3]. Patients’ expectations of a highly successful cataract surgery outcome with additional presbyopia correction are constantly increasing in terms of visual quality and postoperative functional vision, aiming to achieve a total, or nearly total, spectacle independence [4].

For these reasons, the number of IOLs with an augmented range of vision has significantly increased in recent years. There is an increasing focus on emerging technologies aimed at enhancing the intermediate visual outcomes of IOLs, while mitigating the undesired side effects associated with multifocal IOLs [5,6,7,8]. As a result, Extended Depth of Focus (EDOF) IOLs were introduced to improve intermediate visual acuity while minimizing photic phenomena [4,9]. Lately, a new class of monofocal IOLs, named “enhanced monofocal IOLs”, has been developed, providing the same excellent distance vision of a monofocal with improved intermediate vision [10,11,12,13,14].

In this increasingly diverse context, classifying IOLs has become progressively more challenging. Recently, a new evidence-based functional classification has been developed [15,16]. Two principal categories can be distinguished based on the defocus curve’s range of focus (RoF) and its configuration: PARTIAL-RoF and FULL-RoF. Within the PARTIAL-RoF group, three subtypes are defined according to the extent of the RoF achieved: narrow, enhanced, and extended. Conversely, subcategories of FULL-RoF IOLs are classified based on the steepness of the visual acuity (VA) improvement from intermediate to near distances: continuous, smooth transition, and steep transition.

In this new context, the aim of the current study is to compare visual outcomes, optical quality (including optical bench analysis), and patient satisfaction between eyes implanted with a new enhanced monofocal IOL, Evolux (SIFI S.p.A., Catania, Italy), and the standard monofocal IOL, Tecnis GCB00 (Johnson & Johnson Vision Care, Santa Ana, CA, USA). These IOLs are classified as PARTIAL-RoF: PARTIAL-RoF narrow for Tecnis GCB00 and PARTIAL-RoF enhance for Evolux.

## 2. Materials and Methods

### 2.1. Study Design and Patients

A single-center, prospective, non-randomized, comparative clinical trial was conducted at the Eye Clinic, Department of NEUROFARBA, University of Florence, Italy. From June 2023 to July 2024, 100 eyes of 50 study participants were included. Each patient was bilaterally implanted with the same type of intraocular lens. Prior to the study, local ethics committee approval (protocol number Ethics Committee FA000459, ID study 24175) was obtained. In accordance with the tenets of the Declaration of Helsinki, each study participant received a careful explanation of the intended procedure and signed an informed consent form prior to inclusion.

The inclusion criteria for our analysis were: patients with lens opacification and cataract grade greater than II, according to the Lens Opacities Classification System III (LOCS III); and patients aged 60 years or older. The exclusion criteria comprised an axial length greater than 26.0 mm or less than 21.00 mm, corneal astigmatism higher than ±0.75 D, angle Kappa > 0.5 mm, post-surgical refractive outcome exceeding ±0.50 D, prior ocular surgeries (including corneal or refractive procedures), and ocular comorbidities such as uveitis, external or internal infections, diabetes mellitus with retinal complications, pathological miosis, amblyopia, use of alpha-blockers that could cause floppy-iris syndrome, choroidal hemorrhage, keratoconus, corneal endothelial dystrophy, diabetic retinopathy, uncontrolled glaucoma and/or IOP > 24 mmHg, traumatic cataract, pseudoexfoliation syndrome, pupillary abnormalities including aniridia and/or pupillary diameter in photopic conditions ≤ 2.5 mm and in mesopic conditions ≥ 6 mm, microphthalmia, strabismus, nystagmus, pregnancy or lactation period for female patients, degenerative visual disorders (e.g., macular degeneration, optic nerve atrophy, or other retinal disorders).

### 2.2. Patient Evaluation

Prior to surgery, all participants underwent a thorough preoperative ophthalmic evaluation, which encompassed the assessment of monocular and binocular Uncorrected Distance Visual Acuity (UDVA) and best-corrected distance visual acuity (CDVA) analyzed in photopic conditions using 85 cd/m^2^ illumination with 100% contrast with the Early Treatment Diabetic Retinopathy Study (ETDRS) charts at a distance of 4 mt, optical biometry (IOL Master 500, Carl Zeiss Meditec AG, Jena, Germany), subjective and objective refraction, biomicroscopy (Accuref K-900, Shin-Nippon, Tokyo, Japan), Goldmann applanation tonometry, corneal topography and anterior segment optical coherence tomography (AS-OCT) (MS-39, CSO, Florence, Italy), macular OCT (DRI OCT Triton 3D, Topcon Medical Systems, Inc., Oakland, NJ, USA), and dilated fundoscopy. The IOL power and predicted postoperative refraction were based on biometric data (IOL Master 500, Carl Zeiss Meditec, Jena, Germany), calculated using the Barret Formula with an A-constant of 118.35. The IOL power was selected to achieve the predicted postoperative refraction closest to emmetropia. Postoperatively, patients were evaluated at 1 day, 1 month, 3 months, and 6 months. Visual acuity was recorded under photopic conditions and included monocular and binocular UDVA, best-corrected distance visual acuity (BCDVA) at 4 mt, uncorrected intermediate visual acuity (UIVA) and distance-corrected intermediate visual acuity (DCIVA) at 66 cm, and uncorrected near visual acuity (UNVA) and distance-corrected near visual acuity (DCNVA) at 40 cm. Distance visual acuities (UDVA and CDVA) were assessed at 4 m using high-contrast (100%) ETDRS charts illuminated by a calibrated 85 cd/m^2^ light source (Precision Vision, Woodstock, IL, USA). Intermediate and near acuity tests were performed using printed high-contrast ETDRS charts (Precision Vision) at 66 cm and 40 cm, respectively. Photopic lighting conditions were standardized using a halogen lamp with a controlled potentiometer, aligned with ambient brightness values measured by a digital light meter (ST-1300, STANDARD Instruments Co., Ltd., Hong Kong). Defocus curves were acquired binocularly using the subject’s best distance correction. Defocus was simulated by incrementally introducing trial lenses in 0.50 diopter steps, ranging from +2.00 D to −4.00 D, and assessing visual acuity after each step. Binocular contrast sensitivity was tested under photopic conditions using the Optec 6500 Vision Tester (Stereo Optical Co., Inc., Chicago, IL, USA). Objective evaluation of ocular optical quality was carried out using the AcuTarget HD Analyzer (Visiometrics USA, Costa Mesa, CA, USA), a device based on double-pass imaging technology. Measurements were obtained with a 4.0 mm pupil and included the objective scatter index (OSI), modulation transfer function (MTF) cutoff, and the Strehl ratio derived from the point spread function (PSF) [17,18]. Moreover, we measured HOAs at 4.0 mm pupil size using an Osiris-T aberrometer (CSO, Florence, Italy).

At the end of the last follow-up visit, halometry was also performed to assess the presence of lens-induced halos, using the Aston App Halometer [19]. The examination is conducted in a dark room with the patient positioned 2 m away from an LED device located at the center of an iPad. The operator controls the iPad using an iPhone connected via Bluetooth and projects 0.3 LogMAR letters, proceeding from the periphery towards the center of the iPad, where the light source is located, until the patient can recognize the letter. This evaluation is repeated in each of the 8 or 6 directions (at the operator’s discretion) to determine the objective area of obscuration caused by the patient’s halo. For each patient, a halometer glare map was created to represent the halo area.

Effective lens position (ELP) was evaluated at 1, 3, and 6 months using AS-OCT imaging in a single-line scan mode. Patients were seated and instructed to fixate on the internal target. Two images were captured at 0 degrees and 90 degrees, and the image with the highest quality from each pair was selected for analysis. No topical cycloplegic agent was administered during the exams, and all scans were conducted by the same examiner, who was blinded to the IOL model [20]. Finally, the Patient-Reported Spectacle Independence Questionnaire (PRSIQ) was conducted to evaluate spectacle independence during daily life [21].

### 2.3. Intraocular Lenses

The Evolux (SIFI Spa, Catania, Italy) lens is a novel enhanced monofocal IOL, based on a non-diffractive profile, designed to provide better intermediate vision and equivalent distance vision when compared to a standard monofocal IOL. It is a preloaded hydrophobic acrylic IOL with a 6 mm optic diameter and an overall diameter of 13 mm. It is characterized by a biconvex optical design based on positive/negative spherical aberration (SA) distributed in the central 4.5 mm zone of the anterior surface of the IOL, with an aspheric monofocal periphery. It is available in powers ranging from +5.0 D to 30.0 D, with 0.50 D steps. Evolux was developed on a patented technology platform, creating a single elongated focal point to enhance the depth of the field in order to improve intermediate vision.

The Tecnis one-piece GCB00 (Johnson & Johnson Vision Care, Santa Ana, CA, USA) is a standard monofocal IOL. It is an aspherical acrylic hydrophobic IOL with a 6 mm optic and an overall diameter of 13 mm.

### 2.4. Surgery

The surgical interventions were performed by the same highly experienced surgeon (R.M.), all using topical anesthesia. After the creation of a temporal self-sealing corneal incision and a capsulorrhexis of about 5.5 mm, phacoemulsification of the nucleus and irrigation/aspiration were performed (Centurion, Alcon Laboratories Inc., Geneva, Switzerland). The IOL was implanted and, at the end of the surgery, intracameral cefuroxime was injected into the anterior chamber. Routine postoperative treatment included topical nonsteroidal anti-inflammatory, steroidal, and antibiotic eye drops for 2–4 weeks. No intraoperative or postoperative complications occurred.

### 2.5. Statistical Analysis

The data analysis for this study was performed using Stata 18.0 (StataCorp, College Station, TX, USA). To summarize the numerical data, descriptive statistics were applied, including the calculation of mean values and standard deviations. Both groups exhibited normally distributed data, with the assumption of normality assessed using the Shapiro–Wilk test. A two-tailed Student’s *t*-test with 95% confidence intervals was used to compare the clinical characteristics of the two treatment groups. A *p*-value of <0.05 was set as the threshold for statistical significance.

### 2.6. Optical Bench Analysis

The optical characterization of the IOLs was performed with the optical benches PMTF (LambdaX) and NIMO (Lambda X), and then analyzed with our software written in the Matlab programming language. We measured the modulation transfer function (MTF) of the IOLs and the images of the USAF (United States Air Force) resolution target as a function of the defocus using the PMTF in through-focus mode. We also measured the power map using the NIMO optical bench, based on phase-shifting and Schlieren techniques. The MTF measures the degradation of contrast in an image compared to the contrast of the original object, quantifying the image quality of an optical system. USAF is a standard image system used as an evaluation tool to test the resolution. Moreover, the power map represents the spatially resolved power of the IOL.

## 3. Results

The final study sample included 100 eyes from 50 patients who underwent bilateral cataract surgery. Twenty-five patients were bilaterally implanted with Evolux IOL, while the other twenty-five patients received the GCB00 IOL. Cataract surgery in the second eye of the same patient was performed at least 1 month apart. The rate of posterior capsular opacification (PCO) observed at 6 months was 2% (one eye) in both groups. Table 1 shows the patient’s characteristics at inclusion. Both groups of patients exhibited homogeneous preoperative characteristics, with no statistically significant differences. No patients were lost during the follow-up period.

### 3.1. Refractive and Visual Outcomes

Six months postoperatively, all patients reached good levels of UDVA and BCDVA, but the UIVA and DCIVA were significantly better in the Evolux compared to the GCB00 group (*p*-value, respectively, <0.001 and 0.004). Table 2 summarizes visual outcomes for the 2 groups.

### 3.2. ELP

Effective lens position was evaluated at 1 month, 3 months, and 6 months postoperatively with AS-OCT in a single-line scan mode. The ELP, defined as the distance from the central corneal endothelium to the anterior surface of the IOL, was measured using AS-OCT MS-39 (CSO, Florence, Italy) with the aid of caliper function. Table 3 shows that there are no statistically significant differences between the Evolux and GCB00 groups.

### 3.3. Aberrometric Parameters and Optical Quality

Table 4 shows the results of higher-order aberrations (HOAs) evaluated at 4.0 mm pupil size, 6 months postoperatively. All parameters were lower than 0.300 microns, with no significant differences in the two groups. Internal spherical aberration (SA) and internal HOAs showed no statistically significant differences in the two groups (*p*-value 0.098 and 0.124, respectively). Table 4 also shows optical quality parameters measured with the OQAS system at 4.0 mm pupil size. The comparison between the two groups revealed no statistically significant differences in the objective scatter index (OSI), modulation transfer function (MTF) cutoff, or Strehl ratio (*p*-value = 0.179, 0.129, and 0.096, respectively).

### 3.4. Binocular Defocus Curve

**Figure 1:**
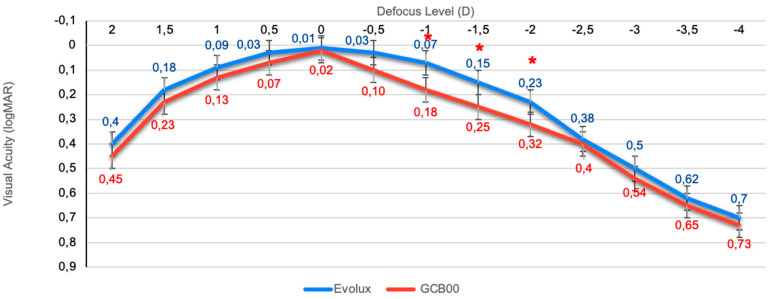
Comparison between mean defocus curves in the IOL groups (logMAR). * *p*-values for −1.00 D, −1.50 D, and −2.0 D are statistically significant (0.038, 0.023, and 0,035, respectively).

The binocular defocus curve, illustrated in Figure 1, showed the highest visual acuity at 0.00 D (4 m) in both groups, with a gradual decline in visual acuity as defocus shifted to both negative and positive values. While the GCB00 IOL exhibited a notable drop in the curve with increasing negative defocus, the Evolux IOL displayed a smoother trend, particularly within the intermediate defocus range (−1 to −2 D), demonstrating superior performance at intermediate distances compared to the GCB00 IOL. *p*-values reached statistical significance at −1.00 D, −1.50 D, and −2.00 D.

### 3.5. Binocular Contrast Sensitivity

**Figure 2:**
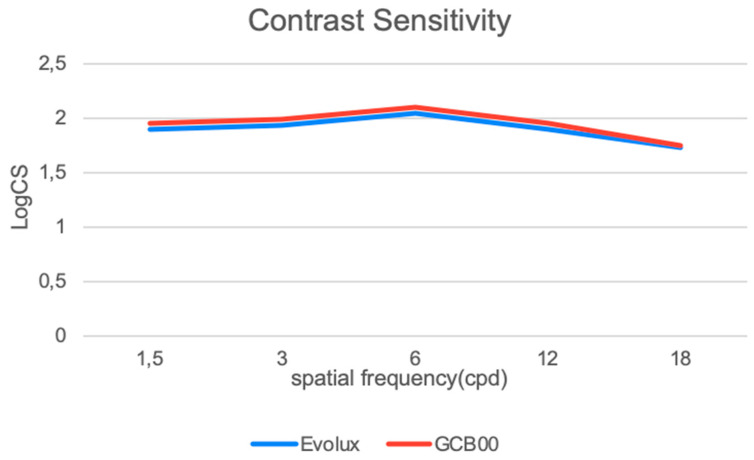
Binocular contrast sensitivity measured with the Optec 6500 Vision Tester under photopic conditions at different spatial frequencies (cycles per degree) (LogCS = Log Contrast Sensitivity).

Figure 2 shows contrast sensitivity (CS) under photopic conditions. No statistically significant differences were found between the GCB00 IOL group and the Evolux IOL group across all spatial frequencies.

### 3.6. Halometry

**Figure 3:**
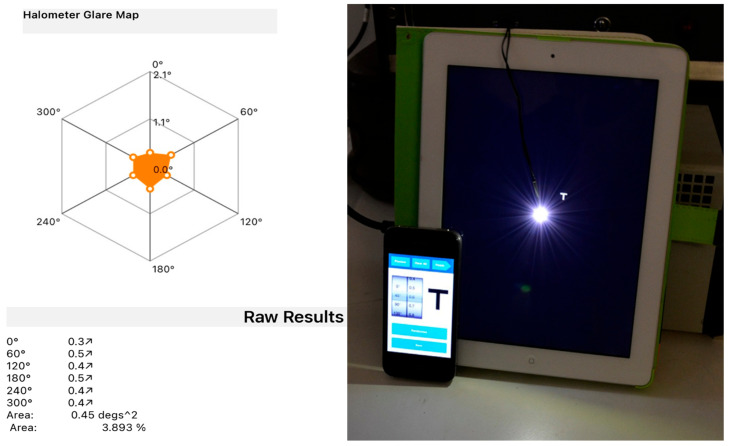
On the left side is the halometer glare map with area expressed in degrees^2^. On the right side is an explanatory image of the halometer.

In terms of halo assessment, the Evolux IOL group achieved an average halo size of 0.44 ± 0.03 degs^2^, while the GCB00 IOL group achieved 0.42 ± 0.04, with a *p*-value of 0.098, which was not statistically significant. Figure 3 shows, on the left side, the result of a single examination with a halometer glare map and a numerical result indicating the corresponding area. On the right side, there is an explanatory image of the instrument.

### 3.7. Optical Bench Analysis Results

**Figure 4:**
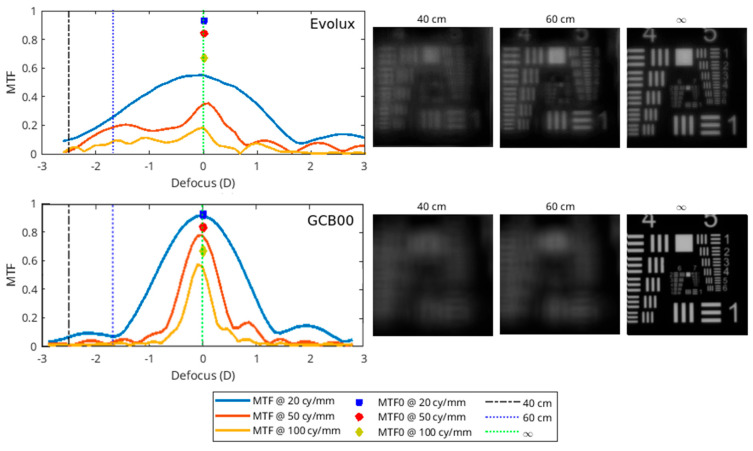
MTF (**left side**) and USAF chart (**right side**) for Evolux IOL (**upper image**) and GCB00 IOL (**lower image**).

In Figure 4, we report the MTF as a function of defocus for three spatial frequencies: 20 cycles/mm (blue line), 50 cycles/mm (red line), and 100 cycles/mm (yellow line). As an upper limit, we also report the pure diffraction case (labelled as MTF0), with blue, red, and yellow markers in the focus. The three dashed vertical lines represent the values of the defocus that correspond to the far (infinite distance), intermediate (60 cm), and near (40 cm) vision. For these same vision distances, the images of the USAF are shown for the two IOLs, providing visual information about the contrast degradation of the image. In both cases, the power of the IOL is 21 D and the pupil size is 3.0 mm. The results show a larger depth of field with Evolux IOL, providing better performance for the near-intermediate vision compared to the GCB00 IOL. The GCB00 IOL instead presents a better contrast preservation in distance vision.

**Figure 5:**
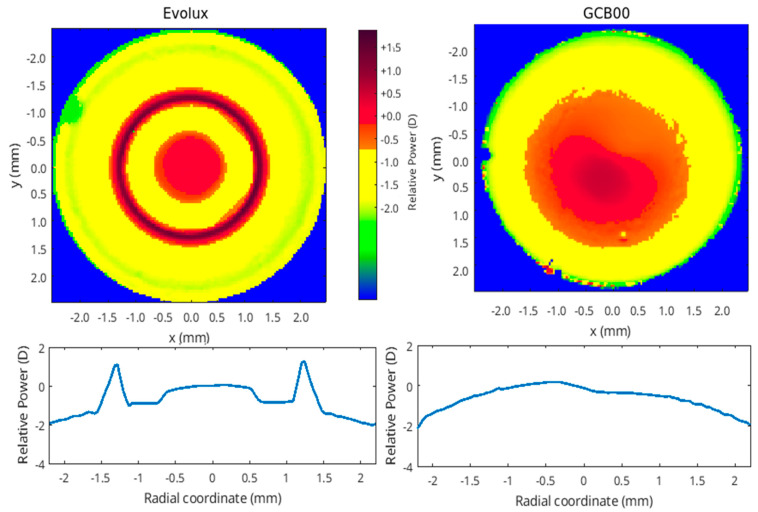
Power Map comparison of Evolux IOL (**left side**) and GCB00 IOL (**right side**). In the lower part of the image, the IOL power is reported as a function of the radial coordinate.

Figure 5 shows the power map of the IOLs with the same power (21 D). The scale is relative to the central value. The characteristics both in shape and in the range of the power show the differences in the two IOLs, in agreement with the optical performances shown in Figure 4. In fact, the large range of power values in the Evolux IOL results in a larger depth of field compared to the GCB00. Furthermore, the power map reflects the optical/geometric design of the two IOLs described in Section 2.3. The GCB00 has a nearly constant power in the central part with a periphery aspheric region with a negative value that can compensate for the natural spherical aberration to obtain a true monofocal lens. The Evolux presents an alternate variation of power in the central part to give an enhanced depth of field that characterizes this IOL. In the lower part of Figure 5, the power is reported as a function of the radial coordinate obtained by averaging two perpendicular meridians.

### 3.8. Questionnaire

The PRSIQ (Patient-Reported Spectacle Independence) assesses the level of spectacle independence in daily activities. None of the patients reported needing spectacles for distance vision. The Evolux IOL group demonstrated greater spectacle independence, particularly for intermediate distances, with only 23% of patients requiring intermediate correction during daily tasks, compared to 87% in the GCB00 IOL group. Notably, question 3 (“How often did you wear glasses for intermediate vision?”) revealed that 70% of patients in the Evolux IOL group reported being able to comfortably perform intermediate tasks without spectacles, whereas no patients in the GCB00 IOL group expressed such satisfaction.

## 4. Discussion

In modern cataract surgery, patients increasingly seek not only improved visual acuity but also greater functional vision and spectacle independence, aiming for a significant enhancement in quality of life. This evolution in patient expectations has fueled advances in IOL technologies, leading to the development of a broad array of options, including bifocal, trifocal, EDOF, and enhanced monofocal IOLs. Multifocal IOLs can provide uncorrected vision across all distances; however, numerous studies have highlighted their drawbacks—particularly reduced contrast sensitivity and increased visual disturbances such as glare and halos [22]. These side effects, which may be exacerbated under certain lighting conditions, remain among the most frequently reported patient complaints in both the early and late postoperative phases [23,24,25]. These limitations, along with the high cost of multifocal lenses, underscore the importance of careful patient selection and have restricted the widespread adoption of these technologies.

In response, enhanced monofocal and extended depth of field IOLs—categorized under partial RoF lenses [16]—have emerged as viable alternatives. The Evolux IOL is a recent entrant in this category, designed to expand the depth of field by employing a 4.5 mm central zone with modulated positive and negative spherical aberrations, thereby supporting both distance and intermediate visual tasks. Though published evidence on the Evolux IOL remains limited [26,27], early reports—including our own—indicate favorable visual outcomes, both corrected and uncorrected, without a significant increase in photic phenomena or decline in contrast sensitivity. Our retrospective, comparative study contributes to this growing body of evidence, demonstrating statistically significant advantages of Evolux over the standard monofocal GCB00, particularly in UIVA and DCIVA. While the UNVA results were unexpectedly strong and better in the Evolux group without being statistically significant, we acknowledge these findings must be interpreted with caution and validated in larger, prospective trials [28]. The defocus curve further supports these results, showing visual acuity better than 0.2 logMAR across a defocus range of +1.00 D to −2.00 D, with a notably smoother performance between −1.00 and −2.00 D in the Evolux group. These findings align with greater functional range at intermediate distances. Moreover, PRSIQ results showed that 70% of patients implanted with Evolux reported independence from spectacles for intermediate tasks. Additionally, the Evolux IOL exhibited favorable aberrometric and contrast sensitivity profiles, comparable to those of GCB00. Our incorporation of halometry offers further value, demonstrating no significant increase in halos among Evolux patients—an objective assessment not previously reported in the literature. Optical bench analysis corroborated the clinical findings, showing greater depth of field for Evolux. Power mapping revealed spatial modulation in the lens center, consistent with its engineered spherical aberration profile. This optical structure directly supports the observed clinical performance in near and intermediate vision. Refractive predictability was high in both groups, with all patients achieving a postoperative SE within ±0.50 D. Evolux eyes showed a mean SE of −0.14 ± 0.31, similar to the GCB00 group. The ELP remained stable throughout follow-up, reflecting the mechanical and material stability of the Evolux IOL.

We acknowledge several limitations of our study. The non-randomized, unmasked design may introduce bias, particularly in subjective outcomes. Additionally, the relatively small and selective sample size may limit generalizability. Pupillary characteristics may also play a role in lens performance—especially for designs like Evolux that rely on central zone modulation—yet this was not directly analyzed in our study. These factors underscore the need for randomized, masked studies with broader inclusion criteria.

In conclusion, the Evolux IOL demonstrated favorable outcomes in intermediate vision. However, these improvements should be interpreted with caution, as the observed benefit at intermediate distances was modest and may be partially attributable to a slight myopic shift. The lens maintained excellent distance vision and demonstrated good overall optical quality, with no significant increase in photic phenomena. Its smooth, non-diffractive design may make it a suitable choice for patients seeking improved visual function across daily tasks without the drawbacks commonly associated with multifocal technologies. Further studies, ideally randomized and with larger populations, are necessary to validate these findings and directly compare Evolux with other PARTIAL-RoF enhanced IOLs.

## Figures and Tables

**Table 1:** Preoperative demographic and clinical characteristics of study participants in the 2 groups.

Characteristics	Evolux IOL	GCB00 IOL	*p*-Value *
Patients (n)	25	25	
Eyes (n)	50	50	
Age (years)	66.3 ± 2.5	65.6 ± 2.1	0.145
AL (mm)	23.32 ± 0.56	24.17 ± 0.63	0.089
CYL (D)	0.48 ± 0.21	0.40 ± 0.33	0.064
SE (D)	1.54 ± 0.73	1.34 ± 0.68	0.076
UDVA (logMAR)	0.48 ± 0.15	0.45 ± 0.09	0.110
BCDVA (logMAR)	0.41 ± 0.12	0.42 ± 0.10	0.097
Anterior chamber depth (mm)	3.02 ± 0.25	3.11 ± 0.18	0.104
IOL power (D)	22.2 ± 2.0	22.0 ± 1.8	0.143

Results are expressed as mean ± standard deviation. AL = axial length; CYL = Cylinder; SE = Spherical Equivalent; UDVA = binocular Uncorrected Distance Visual Acuity; BCDVA = binocular Best Corrected Distance Visual Acuity. * Statistical analysis: Student’s *t*-test. No statistically significant differences were observed between groups (*p* > 0.05).

**Table 2:** Visual performance and subjective refraction 6 months following implantation of Evolux and GCB00 IOLs.

Outcome	Evolux IOL	GCB00 IOL	*p*-Value *	CI
SE (D)	−0.14 ± 0.31	−0.22 ± 0.20	0.098	[0.01; −0.17]
UDVA (LogMAR)	0.03 ± 0.03	0.03 ± 0.02	0.953	[−0.01; 0.01]
BCDVA (LogMAR)	0.01 ± 0.02	0.01 ± 0.02	0.811	[−0.01; 0.01]
UIVA (LogMAR)	0.10 ± 0.06	0.26 ± 0.09	<0.001 *	[−0.21; −0.11]
CIVA (LogMAR)	0.02 ± 0.02	0.03 ± 0.03	0.106	[−0.03; 0.01]
DCIVA (LogMAR)	0.14 ± 0.06	0.20 ± 0.06	0.004 *	[−0.09; −0.02]
UNVA (LogMAR)	0.36 ± 0.12	0.44 ± 0.17	0.071	[−0.16; 0.01]
CNVA (LogMAR)	0.02 ± 0.03	0.05 ± 0.06	0.067	[−0.05; 0.01]
ADD NEAR (D)	1.35 ± 0.43	2.57 ± 0.23	0.143	[0.41; 2.85]

Results are expressed as mean ± standard deviation. CI = Confidence Interval; UIVA = binocular Uncorrected Intermediate Visual Acuity; CIVA = binocular Corrected Intermediate Visual Acuity; DCIVA = binocular Distance-Corrected Intermediate Visual Acuity; UNVA = binocular Uncorrected Near Visual Acuity; CNVA = binocular Corrected Near Visual Acuity. * Statistical analysis: Student’s *t*-test.

**Table 3:** ELP for Evolux and GCB00 IOLs at 1, 3, and 6 months postoperatively.

Postoperative Time	Evolux ELP (mm)	GCB00 ELP (mm)	*p*-Value *
1 month	4.10 ± 0.23	4.03 ± 0.18	0.298
3 months	4.12 ± 0.24	4.08 ± 0.17	0.123
6 months	4.15 ± 0.21	4.10 ± 0.22	0.145

Results are expressed as mean ± standard deviation. * Statistical analysis: Student’s *t*-test. ELP = effective lens position.

**Table 4:** Aberrometric parameters and optical quality results 6 months after implantation of Evolux and GCB00 IOLs.

Characteristics	Evolux IOL	GCB00 IOL	*p*-Value *
Internal HOA (RMS)	0.16 ± 0.03	0.15 ± 0.02	0.098
Internal SA (RMS)	0.08 ± 0.03	0.05 ± 0.04	0.124
OSI	1.39 ± 0.63	1.41 ± 0.51	0.179
MTF cutoff (c/deg)	30.4 ± 7.5	31.0 ± 4.1	0.129
Strehl ratio	0.16 ± 0.03	0.16 ± 0.05	0.096

Results are expressed as mean ± standard deviation. HOA = higher-order aberration, SA = spherical aberration, OSI = objective scatter index, MTF = modulation transfer function. * Statistical analysis: Student’s *t*-test.

## Data Availability

The data presented in this study are available on request from the corresponding author. The data is not publicly available due to ethical reason.
